# Phytochemical Composition, Antioxidant Activity and HPLC Fingerprinting Profiles of Three *Pyrola* Species from Different Regions

**DOI:** 10.1371/journal.pone.0096329

**Published:** 2014-05-05

**Authors:** Dongmei Wang, Fengyuan He, Zhenjiang Lv, Dengwu Li

**Affiliations:** College of Forestry, Northwest A & F University, Yangling, Shaanxi, China; Islamic Azad University-Mashhad Branch, Mashhad, Iran, Iran (Islamic Republic of)

## Abstract

The present study was performed to investigate the variation of phytochemical composition, antioxidant activity and High Performance Liquid Chromatography (HPLC) fingerprinting profiles of three *Pyrola* species. Thirteen samples (eight *P. decorata*, three *P. calliantha* and two *P. renifolia*) were collected from different regions in China. The tannin, hyperoside and quercetin contents of all samples were determined by reverse-phase HPLC and varied within the range 9.77–34.75, 0.34–2.16 and 0.062–0.147 mg/g dry weigh, respectively. Total flavonoid content was evaluated and varied within the range 16.22–37.82 mg/g dry weight. Antioxidant activity was determined by DPPH assay, with IC_50_ ranging from 7.96 to 50.33 µg/ml, ABTS**•^+^** and FRAP assay, within the range 612.66–1021.05 and 219.64–398.12 µmol equiv. Trolox/g, respectively. These results revealed that there were significant variations in phytochemical profiles and antioxidant activity among all samples. Due to the higher phytochemical content and significant antioxidant activity, *P. calliantha* was selected as the most valuable species, and the *P. calliantha* sample from Left banner of Alxa even possessed the strongest antioxidant activity among all the thirteen samples. Futhermore, Emei Mountain was proved to be the most suitable region for producing *P. decorata*. Moreover, in order to further evaluate the diversities and quality of *Pyrola*, HPLC fingerprint analysis coupled with hierarchical cluster and discrimination analyses were introduced to establish a simple, rapid and effective method for accurate identification, classification and quality assessment of *Pyrola*. Thirteen samples were divided into three groups consistent with their morphological classification. Two types of discriminant functions were generated and the ratio of discrimination was 100%. This method can identify different species of *Pyrola* and the same species from different regions of origin. Also, it can be used to compare and control the quality of *Pyrola* and other natural products prepared from them.

## Introduction

The evergreen herbs of the genus *Pyrola* are traditional Chinese medicinal plants. *Pyrola* is mainly distributed in the northern hemisphere in temperate and cold temperate regions around the world with about 30 species. In China, there are 27 *Pyrola* species, mainly distributed in the west and northeast of the area. [1]. *Pyrola* has very high nutritional and medicinal value: it has been widely used in functional foods and supplemental products for treatment of rheumatism, waist pain, knee pain and high blood pressure [2]–[4]. The water extracts of the plant were reported to inhibit the growth of many kinds of human pathogenic bacilli *in vitro*, which were used in refreshing foods [5]. Nowadays, because of its health benefits, *Pyrola* is consumed as a kind of tea called “Lu shou cha” for daily drinking in China [6].

*Pyrola* contains a range of bioactive components, such as flavonoids, phenols, quinones, terpenes, amino acids, etc. [7], [8]. Polyphenolic compounds are especially abundant in *Pyrola*, which may contribute to its high antioxidant activity. A previous study has reported that the ethyl acetate fraction from *P. incarnata* leaves possessed remarkable antioxidant activities, which were almost comparable to the capacities of vitamin C and butylated hydroxytoluene [6]. Another study has also found that the radical scavenging activity of *P. incarnata* from Tahe was very close to that of vitamin C [1]. Taken together, these previous studies indicate that *Pyrola* exhibits powerful antioxidant activities and has potential application in nutraceutical and functional food products for improving human health.

The chemical compositions of *Pyrola* vary greatly since it has various species and extensive distribution. A previous study from our laboratory demonstrated that ecological factors contributed significantly to the contents of active compounds in *Pyrola* samples [9]. Another study from our laboratory indicated that the metal element contents of *P. decorata* varied immensely from region to region [10]. In addition, Zhang et al. reported the great variation of active components in *P. incarnata* from eight sites in northeast China [1]. Different chemical compositions may lead to significant differences in the effectiveness for health problems and the safety related to intake [11]. Therefore, an evaluation of the diversity of *Pyrola* from different sources would be desirable in order to ensure the quality of *Pyrola* and its derived edible products.

The objectives of this study were: (1) to analyze the variation in phytochemical composition and antioxidant activity (DPPH, ABTS and FRAP assays) in three *Pyrola* species (*P. decorata*, *P. calliantha* and *P. renifolia*) collected from different regions in China; (2) to obtain the HPLC fingerprints of *Pyrola* and establish a useful method for identification, classification and quality assessment of *Pyrola* samples using HPLC fingerprint analysis coupled with hierarchical cluster analysis (HCA) and discrimination analysis (DA); and (3) to provide meaningful information for the selection and application of *Pyrola* in both healthcare and the food industry.

## Materials and Methods

### Instrumentation and reagents

HPLC analysis was carried out with an Agilent Series 1260 liquid chromatograph, equipped with a quaternary gradient pump system and a variable-wavelength detector system, connected to a reverse-phase (RP) SB-C 18 column (5 µm, 4.6×250 mm, Agilent, USA). Data collection was performed using ChemStation software (Agilent, USA).

Hyperoside and quercetin were purchased from Sinopharm Chemical Reagent Co. Ltd (Shanghai, China). Tannin was provided by the Tianjin Dongliqu Tianda Chemical Reagent Factory (Tianjin, China). Chromatographic-grade methanol and CH_3_CN were purchased from Tianjin Bodi Chemical Holding Co. Ltd (Tianjin, China). Other chemicals were of analytical reagent grade and were purchased from Tianjin Bodi Chemical Holding Co. Ltd (Tianjin, China). All aqueous solutions were dissolved in deionized water. Stock solutions of tannin, hyperoside and quercetin were prepared in methanol and were diluted to the desired concentration. All solutions were filtered through 0.22 µm nylon filters before use.

### Plant materials

Whole herbs of thirteen samples (eight *P. decorata*, three *P. calliantha* and two *P. renifolia*) were collected from different regions in China (Table 1). All materials were air-dried and powdered, and were stored at −20°C and protected from light until further analysis.

**Table 1:** Three *Pyrola* species (*P. decorata*, *P. calliantha* and *P. renifolia*) collected from different regions in China.

Species	Sample Number	Origin
*P. decorata*	S1	Dianbingchang, Taibai Mountain, SX
	S2	Lujuanliang, Taibai Mountain, SX
	S3	Shanyang, SX
	S4	Ningshan, SX
	S11	Dadian, Taibai Mountain, SX
	S12	Fangyangsi, Taibai Mountain, SX
	S7	Emei Mountain, SC
	S9	Xiabaiyun, Taibai Mountain, SX
*P. calliantha*	S5	Zhongshansi, Taibai Mountain, SX
	S6	Left banner of Alxa, NMG
	S10	Huzhu northern Mountain, QH
*P. renifolia*	S8	Xingshan, HB
	S13	Linjiang, JL

SX: Shaanxi province, China; SC: Sichuan province, China; NMG: Neimenggu province, China; QH: Qinghai province, China; HB: Hubei province, China; JL: Jilin province, China.

### Ethics statement

Specific permissions were not required for the described field sampling studies or for the collection of plant materials. For any locations/activities, no specific permissions were required. All locations where the plants were collected were not privately owned or protected in any way and the field studies did not involve endangered or protected species.

### Preparation of the extracts

Each powdered sample was extracted twice with ten times of 95% ethanol for 2 h in the dark, under nitrogen, at ambient temperature and pressure. All extracts were stored at −20°C in the dark for further use. Extracts were diluted if necessary. All extractions were performed in triplicate.

### RP-HPLC analysis

Sample solutions were thawed, filtered through 0.22 µm membrane filters, and then separated by RP-HPLC to obtain chromatograms. The amounts of three polyphenolic compounds (hyperoside, quercetin and tannin) were quantified using RP-HPLC at ambient temperature. The mobile phase consisted of H_2_O–CH_3_COOH (200∶1) (solvent A) and CH_3_CN (solvent B). The flow rate was 0.8 ml/min. The gradient program was set as follows: 0–8 min, eluent B was kept at 18%; 8–9 min, eluent B was increased from 18% to 20%; 9–17 min, eluent B was kept at 20%; 17–30 min, eluent B was increased from 20% to 34%; 30–35 min, eluent B was increased from 34% to 65%; 35–45 min, eluent B was kept at 65%. The injection volume was 20 µl, and the detect wavelength was 370 nm. Analyses were performed in triplicate.

### Determination of total flavonoid content

The total flavonoid content was determined by reactions of the AlCl_3_ method with some modification [12]. Rutin (4–40 mg/l) was used for calibration of a standard curve (*y* = 13.067*x*+0.0367; *R* = 0.9996) and results were expressed as milligrams rutin equivalent per gram dry weight (DW) of sample. All samples were analyzed in triplicate.

### DPPH radical scavenging activity

The DPPH radical scavenging activity was measured by a slightly modified method [13], [14]. Amounts of 2 ml of the tested samples (10–90 µg/ml) and the positive controls (quercetin and rutin, 1–50 µg/ml) were mixed with 2 ml DPPH solution (200 µM); the final concentration of DPPH was 100 µM. The mixture was shaken vigorously and allowed to stand in the dark for 30 min. The absorbance at 517 nm was measured with a spectrophotometer. All the tests and the controls were repeated in triplicate. DPPH free radical scavenging activity was calculated using the following equation:
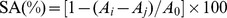
where *A*_0_: absorbance of ethanol (2 ml) and DPPH (2 ml); *A_i_*: absorbance of tested samples (2 ml) and DPPH (2 ml); *A_j_*: absorbance of tested samples (2 ml) and ethanol (2 ml). IC_50_ values were the effective concentrations at which DPPH radicals were scavenged by 50%, and were obtained by interpolation from linear regression analysis.

### ABTS•^+^ radical cation scavenging assay

The method of decolourisation of free radical ABTS•^+^ was performed according to Re et al. with some modification [15]. The ABTS•^+^ was prepared by mixing an ABTS stock solution (7 mM in water) with 2.45 mM potassium persulfate. This mixture was allowed to stand for 12–16 h at room temperature in the dark until reaching a stable oxidative state. For each analysis, the ABTS•^+^ solution was diluted with pH 7.4 phosphate buffered saline (PBS) solution to an initial absorbance of 0.700±0.021 at 734 nm. This solution was freshly prepared for each analysis. For the spectrophotometric assay, 100 µl samples (100 µg/ml) was added to 3.9 ml of ABTS•^+^ solution and the absorbance was determined at 734 nm. Results were expressed in terms of micromoles trolox equivalent per g of dry weight (µmol eq. trolox/g). All determinations were carried out in triplicate.

### Ferric reducing power (FRAP) assay

The method of FRAP assay used was a modified version of that reported by Benzie and Strain [16]. The method is based on the reduction of a colorless ferric complex 2, 4, 6-tripyridyl-s-triazine complex (Fe^3+^-tripyridyltriazine) to a blue-colored ferrous form (Fe^2+^-tripyridyltriazine) by the action of electron-donating antioxidants. The FRAP reagent included 300 mM acetate buffer (3.1 g C_2_H_3_NaO_2_ · 3H_2_O and 1.6 ml C_2_H_4_O_2_), 10 mM TPTZ solution in 40 mM HCl and 20 mM FeCl_3_ · 6H_2_O solution, with the ratio of 10∶1∶1(v/v). For each analysis, 400 µl of sample solutions (500 µg/ml) was added to 3 ml of freshly prepared FRAP reagent. The reaction mixture was incubated for 30 min at 37°C in a water bath in the dark. Then, the absorbance of the samples was measured at 593 nm using the spectrophotometer. The trolox was used as the standard solution. The FRAP results were expressed in terms of micromoles trolox equivalent per g of dry weight (µmol eq. trolox/g). All of the treatment groups were measured in triplicate.

### Data analysis

The correlation coefficients of entire chromatographic patterns among samples were calculated, and the simulated mean chromatograms as well as characteristic peaks were generated using Computer Aided Similarity Evaluation (CASE) software as recommended by the Chinese Pharmacopoeia Committee. The software is used for evaluating similarities between different chromatograms based on the correlation coefficient (median) [17]. The results obtained in this study were calculated using the correlation coefficient unless otherwise specified. HCA and DA were performed using SPSS software (SPSS for Windows 18.0, SPSS Inc., USA) [18]–[20]. The ‘average linkage between groups’ method was applied and the cosine was selected as a measurement [21]. Three principal components obtained by principal component analysis were used to evaluate the similarities and differences among the tested samples [22].

## Results and Discussion

### Validation of the HPLC procedure

Methanol stock solutions containing tannin, hyperoside and quercetin were prepared and diluted to appropriate concentrations for the construction of calibration curves. Six concentrations of each analyte were injected in triplicate, and the calibration curves were constructed by plotting the peak areas under the curve versus the amount of the analytes.

The precision of the analytical method was determined by assaying six replicates of the standard compounds (tannin, hyperoside and quercetin), and the relative standard deviations (RSDs) of the peak areas were estimated to be 0.38–1.16% (*n* = 6). The repeatability of the method was determined by injecting the same sample six times. The areas of the peaks were recorded, and the RSD of the areas varied from 1.13% to 2.49% (*n* = 6). To confirm the accuracy of the method, a recovery experiment was performed by mixing quantified samples with specific quantities of standard compounds. The average percentages of recovery of the three compounds ranged from 97.24±0.02% to 103.51±0.01%. In addition, the RSD varied from 1.13% to 2.20% (*n* = 6). All the results demonstrated that the conditions of the analysis were repeatable and accurate (Table 2).

**Table 2:** Method **v**alidation for the quantitative determination of three compounds using RP-HPLC.

Peak No.	Compounds	Regression equation	Test range (µg/ml)	Precision experiment		Repeatability		Recovery experiment	
				Area of peak	RSD (%)	Area of peak	RSD (%)	Average recovery rate (%)	RSD (%)
1	Tannin	y = 1834.64x+0.8 R^2^ = 0.9999	10–500	362.67	1.16	267.95	1.13	103.51±0.01	1.13
4	Hyperoside	y = 20748.37x–7.83 R^2^ = 0.9997	5–500	1022.23	0.46	174.14	1.98	101.44±0.02	1.83
9	Quercetin	y = 57170.12x–119.2 R^2^ = 0.9998	1–100	2853.37	0.38	14.05	2.49	97.24±0.02	2.20

Each values represented in tables are means ± SD (N = 6).

Three compounds were identified by their retention times (min): tannin (2.34, peak 1), hyperoside (13.26, peak 4), quercetin (32.28, peak 9).

### Content of tannin, hyperoside and quercetin

**Figure 1:**
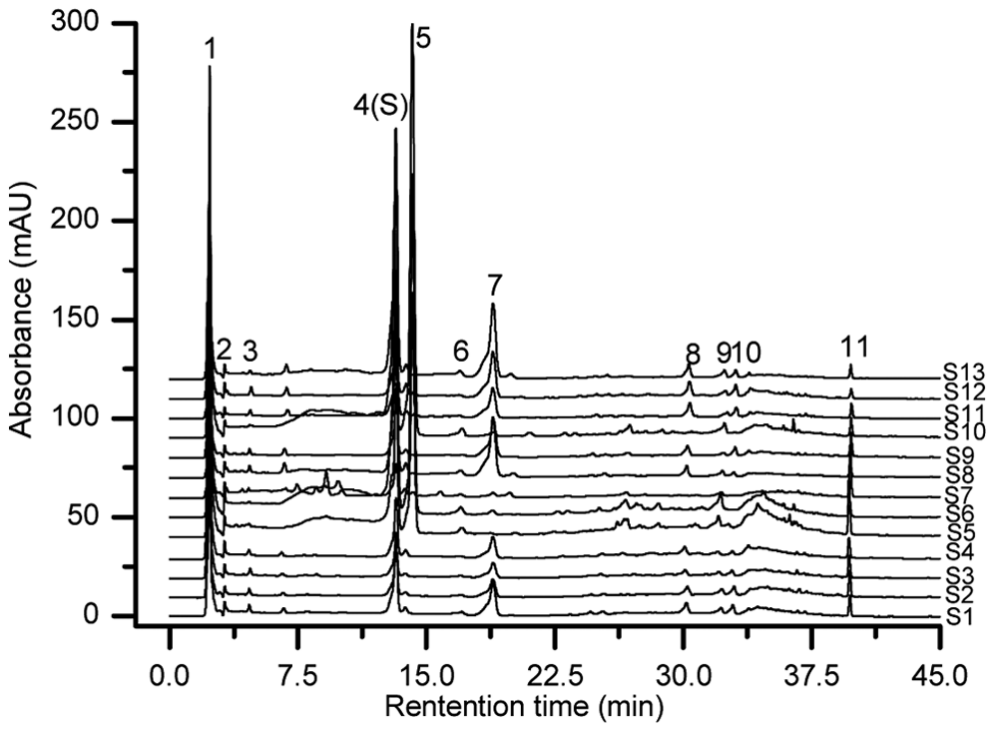
HPLC chromatograms of *Pyrola* samples. Three compounds were identified by their retention times (min): tannin (2.34, peak 1), hyperoside (13.26, peak 4), quercetin (32.28, peak 9).

The contents of tannin, hyperoside and quercetin in three *Pyrola* species from different regions were analyzed by RP-HPLC. Based on a comparison of the retention times with those of the standards, peaks 1, 4 and 9 were identified as tannin, hyperoside and quercetin, respectively (Figure 1). The most abundant phenolic compounds in all samples were tannin and hyperoside, while quercetin was found at lower concentration (Table 3). The contents of tannin, hyperoside and quercetin varied within the ranges 9.77±0.01 to 34.75±0.05 mg/g DW, 0.34±0.01 to 2.16±0.07 mg/g DW and 0.062±0.004 to 0.147±0.007 mg/g DW, respectively. The content of tannin was significantly high in all *Pyrola* samples, especially in *P. calliantha*. Hyperoside was the most abundant in *P. renifolia*. *P. calliantha* had higher contents of these three identified compounds among the three species, and *P. calliantha* from Left Banner of Alxa exhibited the highest content of tannin and quercetin among all samples. For each *P. decorata* sample from eight regions, the content of these three identified compounds varied greatly: the content of tannin, hyperoside and quercetin ranged from 9.77±0.01 to 16.48±0.08 mg/g DW, 0.34±0.01 to 0.96±0.03 mg/g DW and 0.062±0.004 to 0.080±0.006 mg/g DW, respectively. Tannin (16.48±0.08 mg/g DW) and hyperoside (0.96±0.03 mg/g DW) were especially abundant in *P. decorata* from Fangyangsi, while quercetin (0.80±0.006 mg/g DW) was rich in *P. decorata* from Dianbingchang. Recent experimental studies from our laboratory have shown that ecological factors, especially soil factors, contribute significantly to the content of tannin and quercetin in *Pyrola* samples, and this research also found that soil in Fangyangsi is richer in nutrients than other producing regions [8]. This could explain why *P. decorata* from Fangyangsi had high amounts of tannin and quercetin.

**Table 3:** Content of tannin, hyperoside, quercetin and total flavonoids in *Pyrola* samples.

Species	Origin	Content (mg/g DW)			
		Tannin	Hyperoside	Quercetin	Total flavonoids
*P. decorata*	S1	14.26±0.06^bc^	0.86±0.03^cd^	0.080±0.006^bc^	21.42±0.47^cd^
	S2	9.77±0.02^c^	0.46±0.01^e^	0.070±0.008^bc^	17.53±0.72^gh^
	S3	11.84±0.05^c^	0.35±0.01^e^	0.064±0.014^c^	16.22±0.48^h^
	S4	14.90±0.06^bc^	0.55±0.01^de^	0.062±0.004^c^	20.74±0.45^de^
	S11	9.77±0.01^c^	0.82±0.01^cd^	0.063±0.003^c^	20.90±0.44^d^
	S12	16.48±0.08^b^	0.96±0.03^c^	0.076±0.003^bc^	22.84±0.48^c^
	S7	12.62±0.01^bc^	0.34±0.01^e^	0.067±0.001^c^	37.82±0.54^a^
	S9	10.60±0.07^c^	0.57±0.01^de^	0.066±0.002^c^	22.80±0.33^c^
	Average	12.53±2.50^bc^	0.61±0.24^de^	0.069±0.006^c^	22.54±6.61^c^
*P. calliantha*	S5	31.84±0.24^a^	1.79±0.07^b^	0.131±0.006^a^	16.66±0.61^h^
	S6	34.75±0.05^a^	0.46±0.04^e^	0.147±0.007^a^	19.30±0.34^ef^
	S10	20.34±0.12^ab^	1.93±0.03^ab^	0.084±0.002^bc^	18.31±0.51^fg^
	Average	28.98±7.62^a^	1.39±0.81^bc^	0.121±0.033^a^	18.09±1.33^fg^
*P. renifolia*	S8	15.47±0.05^bc^	2.16±0.07^a^	0.074±0.012^bc^	26.77±0.52^b^
	S13	13.12±0.11^bc^	2.09±0.03^a^	0.090±0.003^b^	27.75±0.47^b^
	Average	14.30±1.66^bc^	2.13±0.05^a^	0.082±0.011^b^	27.26±0.69^b^

Each values represented in tables are means ± SD (N = 3).

Values with different letters (a, b, c, d, e, f, g, h) within same column were significantly different (P<0.05).

Tannin, hyperoside and quercetin are all well-known human health antioxidants [23]–[25]. The present results showed that *P. calliantha* was rich in these active components, and could be used as a potential source of antioxidants for the food and drug industries.

### Total flavonoid content

Flavonoids are the most common and widely distributed natural compounds, which are of great interest in nutrition and medicine because of their antioxidant activity and possible protective effects as regards human health [26], [27]. Comparing the three species, *P. renifolia* contained the highest average level of total flavonoid (27.26±0.69 mg/g DW), followed by *P. decorata* (22.54±6.61 mg/g DW) and *P. calliantha* (18.09±1.33 mg/g DW) (Table 3). As to *P. decorata* derived from eight regions, the highest and lowest total flavonoid contents were observed in *P. decorata* from Emei Mountain and Shanyang, respectively. The total flavonoid content of *P. decorata* from Emei Mountain was 37.82±0.54 mg/g DW, which was not only much higher than those of *P. decorata* samples from other regions, but also the highest of all samples. The total flavonoid content of *P. decorata* samples was in the following order: Emei Mountain > Fangyangsi > Xiabaiyun > Dianbingchang > Dadian > Ningshan > Lujuanliang > Shanyang. Based on these results, we may conclude that total flavonoid content varies greatly among different species and among different geographical sources of *Pyrola* samples. Zhang et al. reported that total flavonoid content of *P. incarnata* from different regions varied greatly [1], which could support our findings reported above.

### DPPH radical scavenging activity

DPPH assay has been widely used for the determination of antioxidant activity of pure antioxidant compounds as well as of different plant extracts, a lower DPPH IC_50_ representing stronger antioxidant capacity [10]. For evaluation of antioxidant activity of *Pyrola*, different samples were measured and their DPPH radical scavenging activity compared (Table 4). Among the three species, the highest radical scavenging activity was obtained for *P. calliantha* with the lowest average DPPH IC_50_ value of 9.66±2.09 µg/ml, followed by *P. renifolia* (24.80±2.50 µg/ml) and *P. decorata* (37.11±8.16 µg/ml). DPPH radical scavenging activity of *P. calliantha* collected from Left Banner of Alxa (DPPH IC_50_ = 7.96±0.04 µg/ml) was the highest among all samples, which was better than that of rutin. The DPPH radical scavenging activities of two other *P. calliantha* samples collected from Zhongshansi (DPPH IC_50_ = 9.02±0.03 µg/ml) and Huzhu northern Mountain (DPPH IC_50_ = 11.99±0.06 µg/ml) were also high. It can be concluded that *P. calliantha* may be a valuable antioxidant natural resource.

**Table 4:** Antioxidant activities of *Pyrola* samples.

Species	Origin	DPPH IC_50_ (µg/ml)	ABTS (µmol Trolox/g)	FRAP (µmol Trolox/g)
*P. decorata*	S1	44.56±0.01^g^	612.66±1.26^f^	256.07±0.39^f^
	S2	38.23±0.01^f^	698.52±0.32^e^	223.20±2.31^g^
	S3	27.74±0.02^cd^	735.43±1.65^d^	311.45±2.04^de^
	S4	30.56±0.08^de^	758.05±3.88^d^	306.45±3.94^de^
	S11	42.85±0.11^fg^	643.09±2.54^ef^	223.85±1.85^g^
	S12	50.33±0.01^h^	615.75±0.99^f^	219.64±4.66^gh^
	S7	29.91±0.03^d^	798.23±7.33^c^	309.12±1.47^de^
	S9	32.69±0.14^de^	712.50±0.23^de^	298.09±0.67^e^
	Average	37.11±8.16^f^	696.78±67.94^e^	268.48±42.08^f^
*P. calliantha*	S5	9.02±0.03^b^	958.66±1.45^b^	376.52±2.98^ab^
	S6	7.96±0.04^b^	1021.05±4.21^a^	398.12±2.87^a^
	S10	11.99±0.06^b^	974.20±0.69^b^	355.04±0.61^bc^
	Average	9.66±2.09^b^	984.64±32.48^b^	376.56±21.54^ab^
*P. renifolia*	S8	26.56±0.03^cd^	828.60±9.54^c^	316.45±5.32^d^
	S13	23.03±0.02^c^	809.32±1.27^c^	335.41±1.47^cd^
	Average	24.80±2.50^c^	818.96±13.63^c^	325.93±13.41^d^
Quercetin		2.60±0.04^a^		
Rutin		10.42±0.33^b^		

Each values represented in tables are means ± SD (N = 3).

Values with different letters (a, b, c, d, e, f, g, h) within same column were significantly different (P<0.05).

For *P. decorata* samples from eight regions, the DPPH IC_50_ ranged from 27.74±0.02 µg/ml to 50.33±0.01 µg/ml. The sample from Shanyang had the highest DPPH radical scavenging ability (DPPH IC_50_ = 27.74±0.02 µg/ml). These data indicated that the antioxidant activities of the same *Pyrola* species varied immensely from region to region.

### ABTS•^+^radical cation scavenging activity

ABTS activity was quantified in terms of percentage inhibition of the ABTS•^+^ radical cation by antioxidants in each sample. The ABTS values of the thirteen samples were presented in Table 4. All samples showed the capacity to neutralise the radical cation ABTS•^+^ and showed significant difference (P<0.05). Among the three species, the highest activity was obtained from *P. calliantha* with a value of 984.64±32.48 µmol equiv. Trolox/g, followed by *P. renifolia* and *P. decorata* with values of 818.96±13.63 and 696.78±67.94 µmol equiv. Trolox/g, respectively.

For *P. decorata* samples from eight regions, the ABTS values ranged from 612.66±1.26 to 798.23±7.33 µmol equiv. Trolox/g. The sample from Emei Mountain had the highest ABTS value (798.23±7.33 µmol equiv. Trolox/g).

### Ferric reducing power (FRAP) assay

The FRAP assay evaluated the antioxidant properties of the samples based on their reducing ability. The values obtained from thirteen samples (Table 4) were significantly different (p<0.05). In details, *P. calliantha* possessed the highest antioxidant capacity with a FRAP value of 376.56±21.54 µmol equiv. Trolox/g, followed by *P. renifolia* and *P. decorata* with values of 325.93±13.41 and 268.48±42.08 µmol equiv. Trolox/g, respectively.

As to *P. decorata* derived from eight regions, the FRAP value ranged from 219.64±4.66 to 311.45±2.04 µmol equiv. Trolox/g. The sample from Shanyang had the highest FRAP value (311.45±2.04 µmol equiv. Trolox/g), followed by samples from Emei Mountain and Ningshan with values of 309.12±1.47 and 306.45±3.94 µmol equiv. Trolox/g, respectively.

The ABTS and FRAP values showed the same order of activity observed in the DPPH method. Based on these results, we concluded that *Pyrola* not only presented the highest free radical scavenge capacity but also the strongest reducing capacity. Meanwhile, the antioxidant capacity of *Pyrola* samples varied greatly of different species of *Pyrola* and the same species from different regions of origin.

In conclusion, through comprehensive analysis and evaluation, *P. calliantha* was selected as the most valuable species with the highest average content of total identified compounds (30.49 mg/g DW). It also had the strongest average antioxidant capacity (DPPH IC_50_ = 9.66±2.09 µg/ml, ABTS value = 984.64±32.48 µmol equiv. Trolox/g and FRAP value = 376.56±21.54 µmol equiv. Trolox/g), followed by *P. renifolia* and *P. decorata*. Emei Mountain was proved to be the most suitable region for producing *P. decorata*, because the highest content of combined phytochemicals (total identified compounds and total flavonoids) was observed in the Emei Mountain sample (50.85 mg/g DW). In addition, the antioxidant activity of Emei Mountain sample was the strongest among the eight *P. decorata* samples (DPPH IC_50_ = 29.91±0.03 µg/ml, ABTS value = 798.23±7.33 µmol equiv. Trolox/g and FRAP value = 309.12±1.47 µmol equiv. Trolox/g).

### HPLC fingerprint analysis

Three *Pyrola* species from different regions were analyzed to develop a standard fingerprint under the established HPLC conditions. The simulated mean chromatogram was generated by CASE. Eleven common peaks were selected as characteristic peaks. Based on the comparison of the retention time and UV spectra with standard samples, peaks 1, 4 and 9 were identified as tannin, hyperoside and quercetin, respectively (Figure 1). Peak 4 (hyperoside) was chosen as the reference standard peak due to its peak area accounting for above 10% of the area of all peaks (Table 5). According to the analysis of chromatograms (Figure 1), we could roughly identify different species of *Pyrola* and the same species from different regions of origin. The CASE software was applied to evaluate the similarity of the chromatograms. The results showed that the correlation coefficients of similarity of chromatograms of thirteen samples ranged from 0.24 to 0.99 (Table 6). The wide range of similarity revealed the differences of diverse samples. Furthermore, the similarity of the same species was higher, while that of different species was lower, these results also implying that the chromatograms were representative and associated with phytochemical constituents of *Pyrola* [28].

**Table 5:** The retention time and relative peak area of eleven common peaks of *Pyrola* samples.

Peak No.	Retention time	Relative peak area												
		S1	S2	S3	S4	S5	S6	S7	S8	S9	S10	S11	S12	S13
1	2.34±0.01	1.57	1.99	3.00	2.64	2.10	0.86	3.33	0.62	1.63	1.02	1.23	1.70	0.50
2	3.22±0.01	0.06	0.13	0.12	0.13	0.05	0.02	0.13	0.02	0.07	0.02	0.05	0.06	0.02
3	4.67±0.04	0.05	0.07	0.12	0.04	0.01	0.00	0.04	0.02	0.05	0.00	0.03	0.05	0.01
4	13.26±0.20	1.00	1.00	1.00	1.00	1.00	1.00	1.00	1.00	1.00	1.00	1.00	1.00	1.00
5	13.94±0.26	0.07	0.08	0.09	0.11	1.84	0.80	0.21	0.04	0.09	1.81	0.05	0.15	0.03
6	17.15±0.30	0.06	0.04	0.05	0.02	0.08	0.03	0.10	0.04	0.01	0.05	0.02	0.02	0.02
7	19.01±0.38	0.67	0.62	0.62	0.56	0.01	0.02	0.20	0.48	1.10	0.03	0.70	1.00	0.50
8	30.28±0.23	0.14	0.19	0.16	0.14	0.03	0.00	0.02	0.05	0.18	0.00	0.19	0.17	0.05
9	32.28±0.17	0.08	0.13	0.09	0.07	0.09	0.06	0.14	0.03	0.06	0.06	0.06	0.07	0.03
10	32.95±0.13	0.09	0.15	0.12	0.05	0.02	0.00	0.05	0.02	0.12	0.00	0.10	0.12	0.02
11	39.76±0.05	0.12	0.25	0.27	0.15	0.22	0.01	0.72	0.03	0.20	0.04	0.09	0.06	0.02

Peak 4 (hyperoside) as a reference peak.

**Table 6:** Proximity of the chromatograms of *Pyrola* samples.

No.	S1	S2	S3	S4	S5	S6	S7	S8	S9	S10	S11	S12	S13
S2	0.99												
S3	0.96	0.98											
S4	0.97	0.99	0.99										
S5	0.72	0.74	0.75	0.76									
S6	0.77	0.75	0.72	0.73	0.60								
S7	0.89	0.93	0.95	0.94	0.74	0.70							
S8	0.39	0.42	0.46	0.45	0.36	0.27	0.45						
S9	0.59	0.64	0.69	0.67	0.54	0.41	0.68	0.90					
S10	0.35	0.38	0.41	0.40	0.31	0.25	0.41	0.56	0.53				
S11	0.58	0.63	0.67	0.67	0.50	0.41	0.71	0.35	0.53	0.32			
S12	0.62	0.67	0.72	0.72	0.53	0.46	0.73	0.54	0.79	0.36	0.81		
S13	0.33	0.35	0.38	0.37	0.29	0.24	0.38	0.99	0.87	0.54	0.30	0.50	
SGC	0.85	0.88	0.89	0.89	0.76	0.75	0.88	0.72	0.84	0.65	0.66	0.76	0.67

### Hierarchical clustering analysis

**Figure 2:**
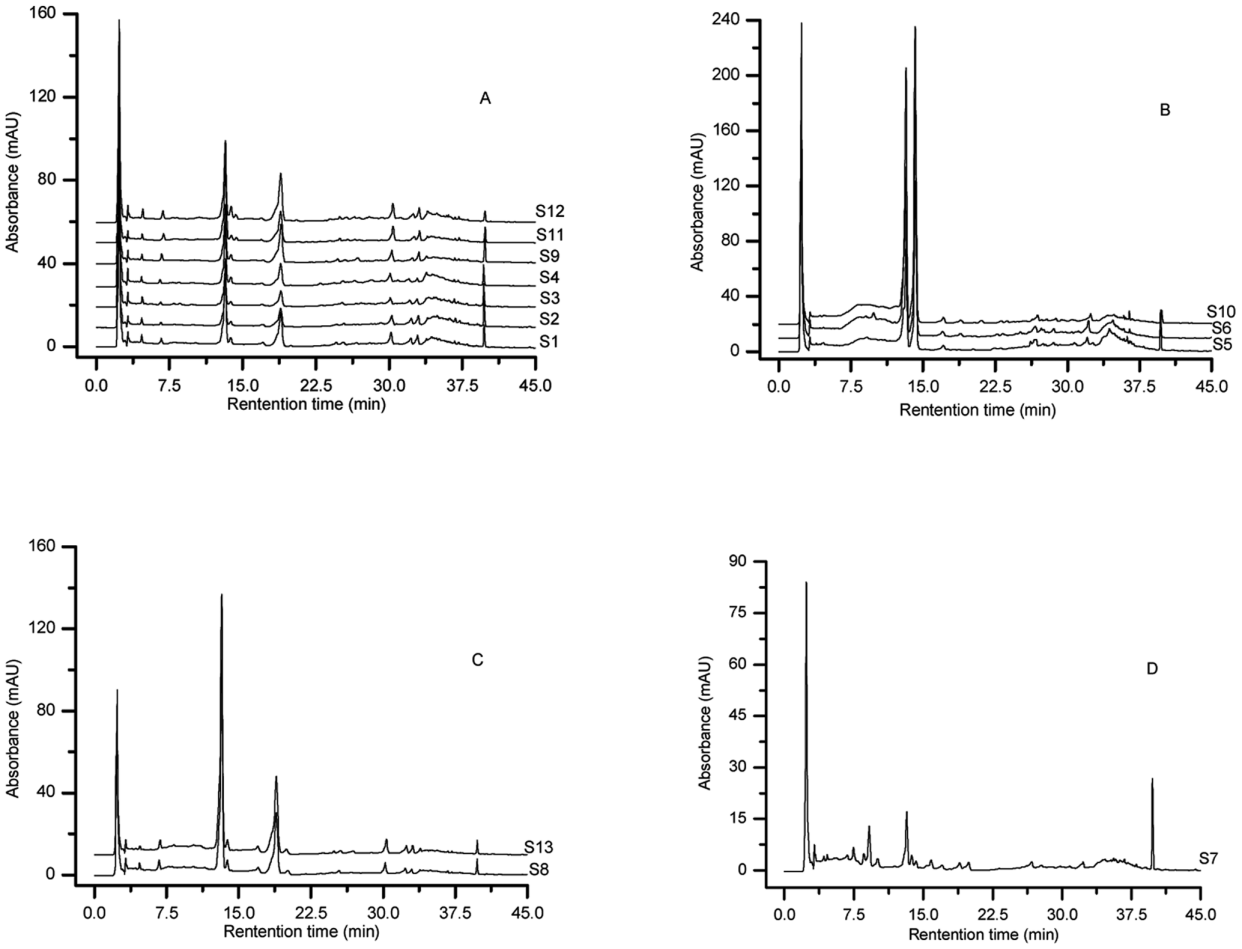
Visual classification of HPLC chromatograms of *Pyrola* samples.

**Figure 3:**
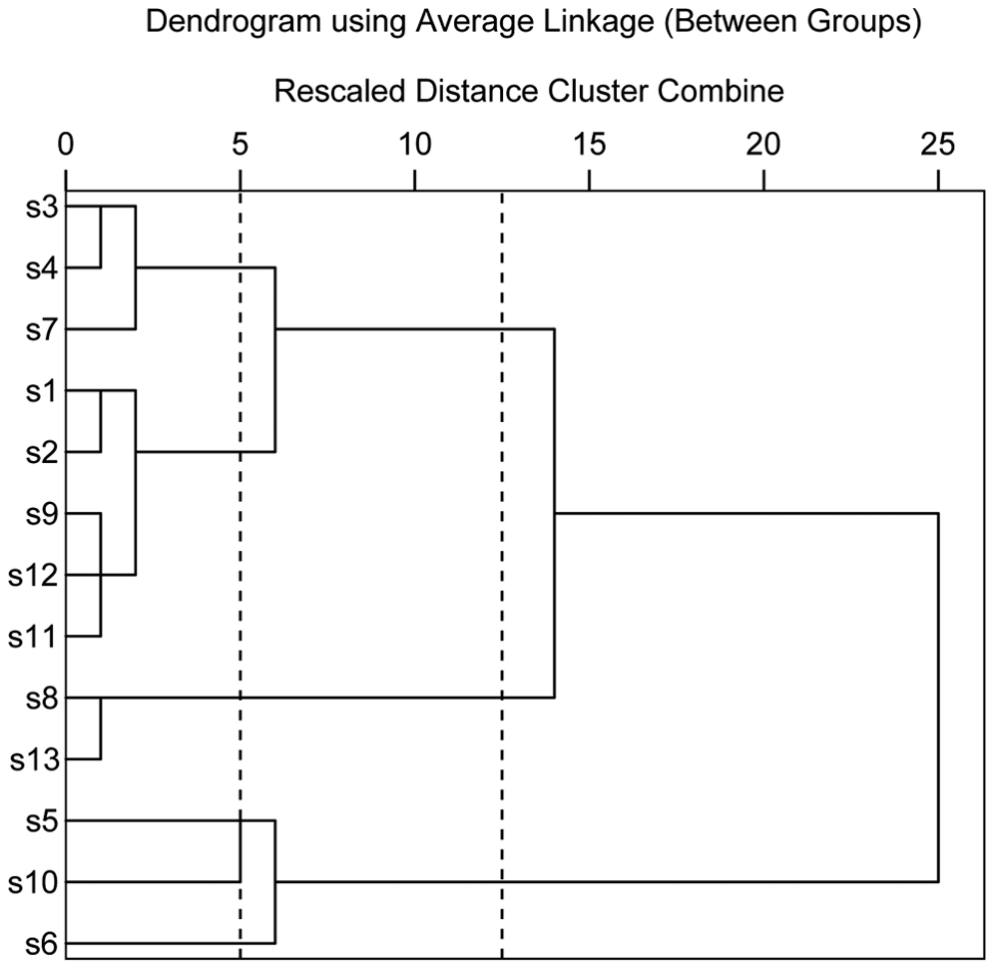
Dendrograms of hierarchical cluster analysis (HCA) for the tested samples of *Pyrola*.

Prior to HCA, we compared the fingerprints visually and simply divided the samples into four distinct groups, namely A, B, C and D (Figure 2). Although it was possible to differentiate chromatograms on the basis of visual comparison, this process was subjective and non-quantitative. HCA can provide a quantitative and objective analysis of fingerprints [20]. According to the chromatograms obtained from all samples, we selected eleven common peaks, using peak 4 as the reference standard peak, and then calculated their relative retention times and relative peak areas. A matrix was applied for HCA using SPSS software, which consisted of the number of the samples and the relative peak area of eleven constituents. A dendrogram was acquired using the average linkage between groups and the cosine method (Figure 3). At a clustering coefficient of 12.5, the thirteen samples can be divided into three groups. Groups A and D were merged into a new class, due to their similar chemical constituents, which revealed the subjectivity of visual classification. The three groups were renamed G1, G2 and G3, which were consistent with the results of morphological classification. At a clustering coefficient of 5, the thirteen *Pyrola* samples were separated into six groups. In other words, HCA not only can distinguish different *Pyrola* species, but also can differentiate the same *Pyrola* species from different regions of origin.

**Figure 4:**
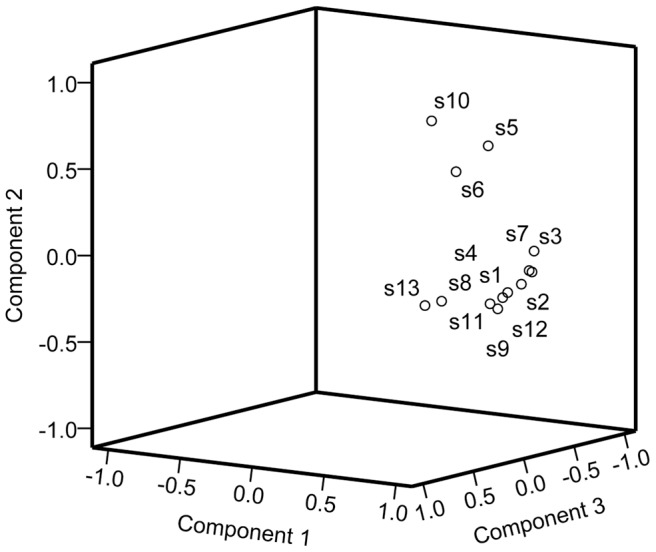
Principal component analysis (PCA) of three principal components for *Pyrola* samples.

The correlation coefficients of each chromatogram for G1, G2 and G3 corresponding to software-generated group simulated mean chromatograms are shown in Table 7. The correlation coefficients between these simulated mean chromatograms are also shown in Table 7. The chromatograms within a particular group were generally consistent. Correlation coefficients for each chromatogram which were classified into a particular group to the software-generated group simulated mean chromatograms were higher than 0.90. However, the chromatograms within a particular group were markedly different from the chromatograms in different groups. Meanwhile, the similarity of the software-generated group simulated mean chromatograms was lower than 0.75 and the difference between groups was evident [20]. Compared with similarities of the different groups, the ANOVN analysis revealed that the difference was significant between groups (*P* = 0.046). In principal component analysis plots (Figure 4), projected points for each group were localized in confined clusters, which can make a good explanation for the HCA results.

**Table 7:** Correlation coefficients between individual chromatograms within a group and the group simulative mean chromatogram, and between the group simulative mean chromatograms.

Group	G1	G2	G3
G1	0.919±0.023	0.661	0.732
G2		0.975±0.005	0.675
G3			0.999±0.000

### Discrimination analysis

DA can be used to build a predictive model of the group membership based on observed characteristics in each case. This procedure can generate a discrimination function or a set of discrimination functions which can discriminate and classify the unknown membership on the basis of eigenvalues [20].

The relative peak areas of eleven common peaks were selected from the chromatograms. However, some peaks were meaningless for establishing the discrimination function. Therefore, DA was applied to selected variables which were of value for the functions, and then the discrimination function was generated. Two types of discrimination function were obtained using the SPSS software.

Canonical discrimination function:
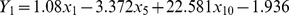


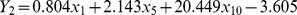



Discrimination standard:
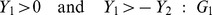


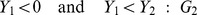


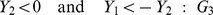



Fisher's discrimination function:
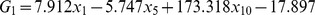


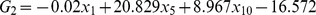


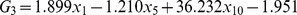



Discrimination standard: there were dependent variables, G1, G2 and G3, which denoted the samples from groups G1, G2 and G3, respectively. *x* represented the independent variable (Table 8). We could obtain three functional values of each sample, and a sample was assigned to the group corresponding to the highest functional value.

**Table 8:** Statistics of discriminant analysis.

Sample number	Actual group	Highest group					Second highest group		
		Predicted group	P(D>d | G = g)		P(G = g | D = d)	Squared mahalanobis distance to centroid	Group	P(G = g | D = d)	Squared mahalanobis distance to centroid
			df	p					
1	1	1	0.653	2	1.000	0.854	3	0.000	180.687
2	1	1	0.928	2	1.000	0.149	3	0.000	179.431
3	1	1	0.165	2	1.000	3.605	3	0.000	177.068
4	1	1	0.757	2	1.000	0.557	3	0.000	174.232
5	2	2	0.988	2	1.000	0.025	1	0.000	15722.999
6	2	2	0.989	2	1.000	0.022	1	0.000	15785.070
7	1	1	0.539	2	1.000	1.235	3	0.000	198.501
8	3	3	0.519	2	1.000	1.312	1	0.000	144.230
9	1	1	0.625	2	1.000	0.939	3	0.000	196.046
10	2	2	0.998	2	1.000	0.004	1	0.000	15770.854
11	1	1	0.042	2	1.000	6.321	3	0.000	117.066
12	1	1	0.160	2	1.000	3.666	3	0.000	159.914
13	3	3	0.519	2	1.000	1.312	1	0.000	199.798

**Figure 5:**
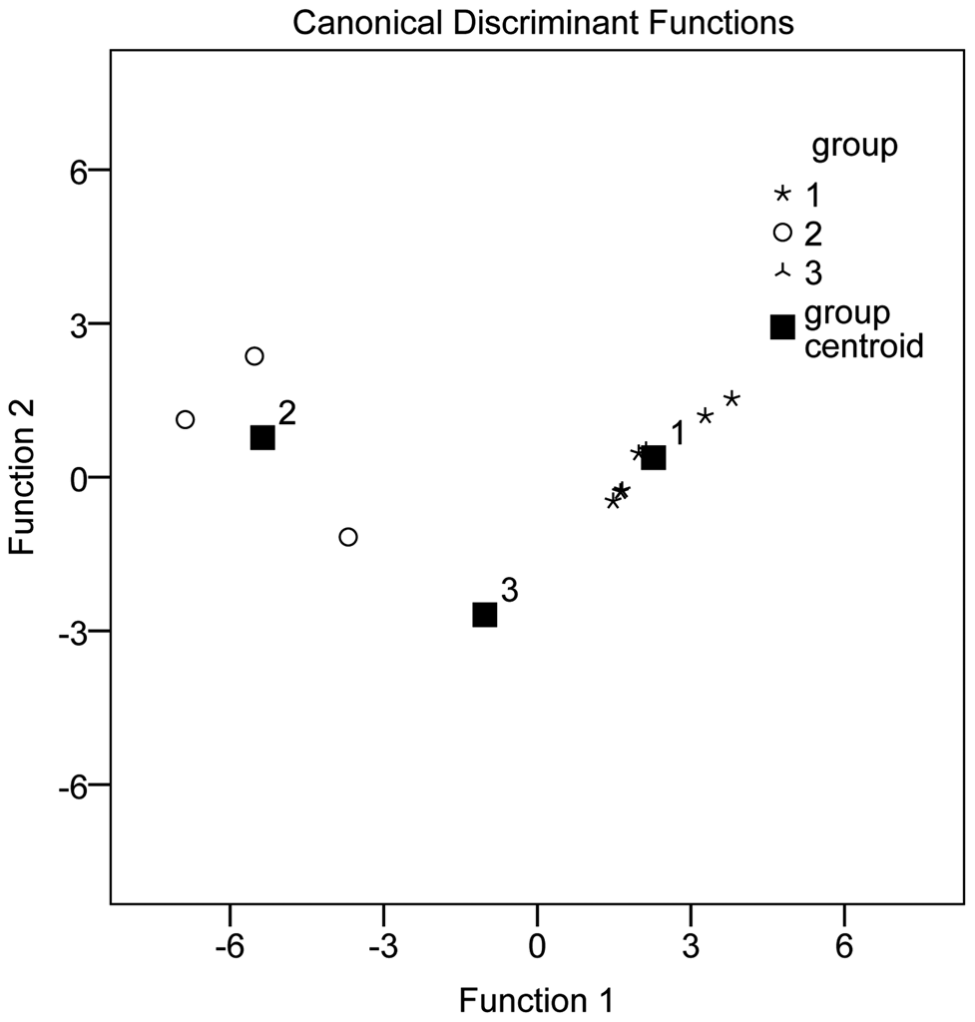
Canonical discrimination analysis (DA) of HPLC chromatograms for *Pyrola* samples.

There were three variables which were selected to constitute the discrimination functions. When we needed to place an unknown sample, we inserted the relative peak areas of the three variables into the equations, and the unknown sample was grouped according to the discriminant standard value obtained. The well-resolved DA plots for the three groups are shown in Figure 5. Using the three most discriminating variables enabled tested samples belonging to groups G1, G2 and G3 to be classified with 100% accuracy.

According to the HPLC fingerprint analysis, the chemical compositions vary greatly among either different species or different geographical sources of *Pyrola* samples. This variation may lead to significant differences in effectiveness as functional foods and nutritional supplements. So it is important to evaluate the quality of *Pyrola*. HPLC fingerprint analysis coupled with HCA and DA in the present research performed well for the quality evaluation of *Pyrola* samples.

## Conclusions

The results from the present study indicated that all tested *Pyrola* samples significantly differed from each other in their phytochemical profiles and antioxidant property. Through comprehensive analysis and evaluation, *P. calliantha* was selected as the most valuable species, and the *P. calliantha* sample from Left banner of Alxa even possessed the strongest antioxidant activity among all the thirteen samples. Futhermore, Emei Mountain was proved to be the most suitable region for producing *P. decorata*. The results indicated that there were rich variations in *Pyrola* samples.

Different chemical compositions may lead to significant differences in effectiveness as functional foods and nutritional supplements. In order to further evaluate the diversities and quality of *Pyrola*, HPLC fingerprint analysis coupled with HCA and DA were introduced to establish a simple, rapid and effective method for the accurate identification, classification and quality assessment of *Pyrola*. Thirteen samples were separated into three groups using HCA at a rescaled distance of 12.5, this separation being consistent with the results of morphological classification. Furthermore, the thirteen samples were separated into six groups at a rescaled distance of 5, which can be identified as the same species of *Pyrola* from different regions of origin. Two types of discriminant functions were generated using three selected predictor variables and the ratio of discrimination was 100%. In summary, this method can identify different species of *Pyrola* and the same species from different regions of origin.

The present study provides meaningful information for the collection and application of *Pyrola* in both healthcare and the food industry. Moreover, the results from this study could be used to compare and control the quality of *Pyrola* and other natural products prepared from them. It is well known that the species of *Pyrola* are very abundant; in this research we only studied three of them, so a large, systematic study of *Pyrola* from different sources is required in a future study. Additionally, it is understood that the DPPH scavenging capacity assay, ABTS•^+^ radical cation scavenging assay and ferric reducing power (FRAP) assay used in the present study may not fully reflect the antioxidative mechanisms of *Pyrola in vivo* and its health promotion properties in the human body, and further cellular and *in vivo* studies of the biological activities are required.

## References

[1] Zhang DY, Luo M, Wang W, Zhao CJ, Gu CB, et al (2013) Variation of active constituents and antioxidant activity in *pyrola* [*P. incarnata* Fisch.] from different sites in Northeast China. Food Chem 141: 2213–2219. 23870950 10.1016/j.foodchem.2013.05.045

[2] Chinese Pharmacopoeia Committee (2005) Pharmacopoeia of China. Beijing: Chemical Industry Press. (In Chinese).

[3] Yamashita CI, Saiki M, Vasconcellos MBA, Sertie JAA (2005) Characterization of trace elements in medicinal plant by neutron activation analysis. Appl. Radiat. Isot 63: 841–846. 10.1016/j.apradiso.2005.05.045 16099665

[4] Liu MG, Xiao GGS, Rong PJ, Zhang ZG, Dong J, et al (2012) Therapeutic effects of *radix dipsaci*, *pyrola* herb, and *cynomorium songaricum* on bone metabolism of ovariectomized rats. BMC Complement Altern Med 12: 67–85. 22639966 10.1186/1472-6882-12-67 PMC3585854

[5] Lou DQ, Yang YZ, Song L, Wang JX (2004) Advances in studies on special plants of *Pyrola* L. in China. Chin. Tradit. Herbal Drugs 35: 463–466 (In Chinese).

[6] Yao XH, Zhang DY, Zu YG, Fu YJ, Luo M, et al (2013) Free radical scavenging capability, antioxidant activity and chemical constituents of *Pyrola incarnata* Fisch. leaves. Ind. Crops Prod 49: 247–255.

[7] Bergeron C, Marston A, Antus S, Gauthier R, Hostettmann K (1998) Flavonoids from *pyrola elliptica* . Phytochem 49: 233–236.

[8] Leonid RP, Kenzo N, Ilya VS, Anna BR (2011) The 1, 4-naphthoquinone derivative from *Pyrola rotundifolia* activates AMPK phosphorylation in C2C12 myotubes. Fitoterapia 82: 1285–1289. 21958969 10.1016/j.fitote.2011.09.005

[9] Lv ZJ, Wang DM, Li DW (2013) Content of four chemical elements and the forms of their existence in *Pyrola decorata* occurring in different areas. Journal of Northwest Forestry University 28 (1): 126–129 (In Chinese with English abstract.).

[10] Lv ZJ, Wang DM, Li DW (2012) Correlation between quality of *Pyrola decorata* and its ecological factors based on hierarchy-vector analysis. Chinese Journal of Plant Ecology 36 (9): 992–1003 (In Chinese with English abstract.).

[11] Zhao Y, Xie ZH, Niu YG, Shi HM, Chen P, et al (2012) Chemical compositions, HPLC/MS fingerprinting profiles and radical scavenging properties of commercial *Gynostemma pentaphyllum* (Thunb.) Makino samples. Food Chem 134: 180–188.

[12] Jia ZS, Tang MC, Wu JM (1999) The determination of flavonoid contents in mulberry and their scavenging effects on superoxide radicals. Food Chem 64: 555–559.

[13] Williams WB, Cuvelier ME, Berset C (1995) Use of a free radical method to evaluate antioxidant activity. Lebensm.-Wiss. u. -Technol 28: 25–30.

[14] Wang SS, Wang DM, Pu WJ, Li DW (2013) Phytochemical profiles, antioxidant and antimicrobial activities of three *Potentilla* species. BMC Complement Altern Med 13: 321–331. 24252124 10.1186/1472-6882-13-321 PMC3840622

[15] Re R, Pellegrini N, Proteggente A, Pannala A, Yang M, et al (1999) Antioxidantactivity applying an improved ABTS radical cation decolorization assay. Free Radical Bio Med 26: 1231–1237. 10381194 10.1016/s0891-5849(98)00315-3

[16] Benzie IF, Strain J (1996) The ferric reducing ability of plasma (FRAP) as a measure of “antioxidant power”: the FRAP assay. Anal Biochem 239: 70–76. 8660627 10.1006/abio.1996.0292

[17] Li BY, Hu Y, Liang YZ, Xie PS, Du YP (2004) Quality evaluation of fingerprints of herbal medicine with chromatographic data. Analytica Chimica Acta 514: 69–77.

[18] Yang DF, Liang ZS, Duan QM, Zhang YJ (2007) Quality assessment of *Cardiotonic Pills* by HPLC fingerprinting. Chromatographia 66: 509–514.

[19] Alonsosalces RM, Guyot S, Herrero C (2005) Chemometric classification of Basque and French ciders based on their total polyphenol contents and CIELab parameters. Food Chem 91: 91–98.

[20] Shi XM, Zhang JS, Tang QJ, Yang Y, Hao RX, et al (2008) Fingerprint analysis of Lingzhi (Ganoderma) strains by high-performance liquid chromatography coupled with chemometric methods. World J. Microbiol. Biotechnol 24: 2443–2450.

[21] Zhao KJ, Dong TTX, Tsim KWK (2003) Molecular genetic and chemical assessment of *Radix Angelica* (Danggui) in China. J Agric Food Chem 51: 2576–2583. 12696940 10.1021/jf026178h

[22] Peng L, Wang YZ, Zhu HB, Chen QM (2011) Fingerprint profile of active components for *Artemisia selengensis Turcz* by HPLC–PAD combined with chemometrics. Food Chem 125: 1064–1071.

[23] Harwood M, Danielewska-Nikiel B, Borzelleca J, Flamm G, Williams G, et al (2007) A critical review of the data related to the safety of quercetin and lack of evidence of *in vivo* toxicity, including lack of genotoxic/carcinogenic properties. Food Chem Toxicol 45(11): 2179–2205. 17698276 10.1016/j.fct.2007.05.015

[24] Yang LC, Li R, Tan J, Jiang ZT (2013) Polyphenolics composition of the leaves of *Zanthoxylum bungeanum* Maxim. grown in Hebei, China, and their radical scavenging activities. J Agri Food Chem 61(8): 1772–1778. 10.1021/jf3042825 23383696

[25] Robards K, Prernzler PD, Tucker G, Swatsitang P, Glover W (1999) Phenolic compounds and their role in oxidative processes in fruits. Food Chem 80: 561–566.

[26] Heim KE, Tagliaferro AR, Bobilya DJ (2002) Flavonoid antioxidants: chemistry, metabolism and structure- activity relationships. Nutri Biochem 13: 572–584. 10.1016/s0955-2863(02)00208-5 12550068

[27] Chantal B, Andrew M, Sandor A, Robert G, Kurt H (1997) Flavonoids from *Pyrola elliptica* . Phytochem 49: 233–236.

[28] Yang J, Chen LH, Zhang Q, Lai MX, Wang Q (2007) Quality assessment of *Cortex cinnamomi* by HPLC chemical fingerprint, principle component analysis and cluster analysis. J. Sep. Sci 30: 1276–1283. 10.1002/jssc.200600389 17623468

